# Research Ethics in the European Influenzanet Consortium: Scoping Review

**DOI:** 10.2196/publichealth.9616

**Published:** 2018-10-10

**Authors:** Lester Darryl Geneviève, Tenzin Wangmo, Damien Dietrich, Olivia Woolley-Meza, Antoine Flahault, Bernice Simone Elger

**Affiliations:** 1 Institute for Biomedical Ethics University of Basel Basel Switzerland; 2 Department of Radiology and Medical Informatics Geneva University Hospitals Geneva Switzerland; 3 Institute of Global Health University of Geneva Geneva Switzerland; 4 ETH Zurich Swiss Federal Institute of Technology Zurich Switzerland; 5 University Center of Legal Medicine University of Geneva Geneva Switzerland

**Keywords:** communicable diseases, influenza, human, public health surveillance, research ethics, web-based technologies, mobile phones, smartphone, participatory surveillance

## Abstract

**Background:**

Influenzanet was launched in several European countries to monitor influenza-like illness during flu seasons with the help of volunteering participants and Web-based technologies. As in the case of developing fields, ethical approaches are not well developed in the collection, processing, and analysis of participants’ information. Existing controversies and varying national ethical regulations can, thus, hamper efficient cross-border research collaboration to the detriment of quality disease surveillance.

**Objective:**

This scoping review characterizes current practices on how ethical, legal, and social issues (ELSIs) pertinent to research ethics are handled by different Influenzanet country groups to analyze similarities and identify the need for further harmonization of ethical approaches.

**Methods:**

A literature search was carried out on PubMed, Web of Science, Global Digital Library on Ethics, and Bioethics Literature Database to identify ELSIs for Influenzanet country platforms. Only English-language papers were included with publication dates from 2003 to 2017. Publications were screened for the application of bioethics principles in the implementation of country platforms. Additional publications gathered from the Influenzanet Consortium website, reference screening, and conference proceeding were screened for ELSIs.

**Results:**

We gathered 96 papers from our search methodology. In total, 28 papers that mentioned ELSIs were identified and included in this study. The Research Ethics Committee (REC) approvals were sought for recruiting participants and collecting their data in 8 of 11 country platforms and informed e-consent was sought from participants in 9 of 11 country platforms. Furthermore, personal data protection was ensured throughout the Consortium using data anonymization before processing and analysis and using aggregated data.

**Conclusions:**

Epidemics forecasting activities, such as Influenzanet, are beneficial; however, its benefits could be further increased through the harmonization of data gathering and ethical requirements. This objective is achievable by the Consortium. More transparency should be promoted concerning REC-approved research for Influenzanet-like systems. The validity of informed e-consent could also be increased through the provision of a user friendly and standard information sheet across the Consortium where participants agree to its terms, conditions, and privacy policies before being able to fill in the questionnaire. This will help to build trust in the general public while preventing any decline in participation.

## Introduction

Web-based technologies have become an integral part of public health surveillance over the last 2 decades [1]. It is estimated that 4.3 billion people globally will have mobile broadband subscriptions by the end of 2017 [2]. Their ubiquitous availability allows volunteer citizens to engage in disease detection through digital means [3]. Real-time granular health data are, thus, collected from volunteering participants (eg, via mobile phones with global positioning), supplementing the big data collected by public health authorities and laboratories [3,4]. Combining these different data sources allows earlier and finer spatial detection of public health threats than traditional surveillance systems, permitting more appropriate preventive and mitigating measures to be deployed [4]. A successful example of such disease digital detection is the European Influenzanet Consortium.

Every year in Europe, seasonal flu brings its share of morbidity and mortality among vulnerable groups (eg, the elderly) and a rise in associated medical costs [5]. Infection with influenza virus is hard to diagnose without virological confirmation, and public health authorities usually rely on influenza-like illness (ILI) as a surveillance indicator for outbreaks [6]*.* This surveillance program is carried out by the European Influenza Surveillance Network (EISN), which is coordinated by the European Centre for Disease Control [7]. Since 2008, EISN has relied on ILI reports by general practitioners from national sentinels in its 30 European Union and European Economic Area countries [8]. However, this traditional surveillance system is biased by the use of nonuniform case definitions for ILI by the Member States and depends on the rate at which patients seek medical care from general practitioners, thereby reflecting only medically attended ILI incidence rates [9-11]. The general practitioner consultation rate is itself dependent on several factors that include the time delay between the onset of symptoms and health complications, the need of certificates from general practitioners for prolonged work absenteeism owing to illness, types of health insurance, and health care systems [12]. Thus, there is a non negligible underestimation of the real disease burden of influenza outbreaks [13]. The current limitations of EISN led to the development of Influenzanet, an innovative ILI surveillance network based on the active participation of public volunteers and the use of Web-based technologies to report cases of ILI, complementing data gathered by EISN [12,14].

The Influenzanet Consortium was launched in 2003 in the Netherlands and the Flemish part of Belgium [6]. Denmark, France, Ireland, Italy, Portugal, Spain, Sweden, Switzerland, and the United Kingdom have also joined this surveillance network [15]. However, very recently in 2017, the Netherlands-Belgium platforms have ceased their activities because of lack of funding, which undermines the excellent work done by these platforms in promoting the ILI surveillance for 15 years in their countries. The platforms will resume their activities if funding is made available by May 2018. Otherwise, the platforms will terminate their activities permanently [16]. Public volunteers are usually recruited via mass media, and there are no exclusion criteria for registration (except for Sweden) [17-19]. At registration, volunteers fill in an intake questionnaire and afterwards, receive a weekly reminder by email to fill in an ILI-related symptom questionnaire [6]. Importantly, the absence of symptoms is also declared. In addition, participants are allowed to indicate symptoms of ILI for other household members in an attempt to increase the data collection for children and elderly individuals [17].

Influenzanet offers numerous advantages over EISN, including the following: ILI incidence rates are extrapolated from both medically attended and unattended patients, real-time disease-monitoring capability through the citizen participation, flexibility to changes without disturbance of the overall system functionality, uniform data collection allowing direct comparisons between countries, comparatively lower running costs, easier to increase scalability, and participant empowerment through information on prevention strategies and disease activity at local and national levels [11,13,17,20].

Nonetheless, Influenzanet also has some disadvantages, including the self-selection bias of participants (eg, underrepresentation of younger and older age groups), absence of virological confirmation of influenza cases, recruitment and motivation of participants to continuously donate their data for surveillance are problematic (eg, limited sample sizes in some countries), and limited amount and complexity of data that can be collected to ensure the continued use of platforms by participants [11,13,17].

This approach for monitoring disease involves the collection of information about users that affects their risks of influenza or complication from influenza; this information includes the demographic data, vaccination status, presence of certain medical conditions (eg, diabetes mellitus), use of food complements, daily activities, and household composition (eg, presence of children) [21]. Moreover, some national platforms have developed mobile apps as additional data collection tools; for instance, the Swiss mobile app gathers useful supplementary data through smartphone sensors (eg, inbuilt movement sensors to test for an association between the physical activity level of participants and the risk of ILI) or smartphone features (eg, test for an association between the psychological profile of participants, inferred via the list of apps installed, and attitude toward vaccination; Grippenet Switzerland, email communication, November 29, 2017). This complements the data gathered from questionnaires, allowing to answer innovative research questions and implement rational public health strategies while maintaining high privacy protection measures; for example, only highly aggregated and summarized versions of the data are transmitted for analysis, whereas the bulk of data is stored locally on participants’ smartphones. Moreover, mobile app data will not be shared with the rest of the Consortium until a framework for data sharing is set up. Nonetheless, Influenzanet data can be valuable and sensitive information. Thus, collecting such information poses ethical, legal, and social issues (ELSIs), in particular, if the collected data could be used for secondary research purposes or in the event of a cyber attack leading to data leakage.

As it is commonly the case for developing fields, ethical approaches are not yet well developed in the collection (here through Web-based technologies), processing, and analysis of participants’ information. Existing controversies can, thus, be the cause of additional barriers to efficient collaboration. Furthermore, although research collaboration and comparability of data are important because epidemics do not stop at national borders, varying ethical regulations at national levels can hamper collaboration between countries. Additionally, a number of new ethical issues raised by Influenzanet-like activities do not or only partially fit traditional evaluation categories used by Research Ethics Committees (RECs) for clinical trials or data-based research. This scoping review aims to discuss ELSIs of these participatory surveillance systems. First, we characterized the current practices using findings from the literature search where we compared how issues related to research ethics are being handled by different Influenzanet country groups to analyze similarities and identify the need for further harmonization of ethical approaches. Thereafter, we carried out an ELSI analysis to suggest ways to strengthen them to lay the ground for expanding the capacity and positive impact of such systems in the future.

## Methods

For this review, we followed the methodological guidance provided for conducting a scoping review [22]. Four databases, namely, PubMed, Web of Science (all databases), Global Digital Library on Ethics, and BELIT (Bioethics Literature Database) were searched to identify ELSIs for the national platforms of the Influenzanet Consortium.

We used the following key terms: *Influenzanet*, *De Grote Griepmeting*, *Flusurvey*, *Gripenet*, *Grippenet*, *Hälsorapport*, *Influmeter*, and *Influweb*. Only English-language papers with publication dates from the last 15 years (2003-2017) were included. The search started from 2003 because the first Influenzanet national platform, *De Grote Griepmeting*, was launched in this year.

In line with the conception of modern research ethics, we searched publications related to Influenzanet for the use and application of ethical principles [23,24] in the implementation of these national platforms; these principles are as follows: respect for autonomy (respect the decision-making capacity of Influenzanet participants through the provision of a: an informed consent and b: opt-out option); beneficence (direct and indirect benefits provided to Influenzanet participants via Web-based information on the study); nonmaleficence (prevention of informational harm to Influenzanet participants such as personal data protection measures, for example, anonymization of personal data); and justice (ensuring open and nondiscriminative participation of users to the Influenzanet network to ensure fairness in the distribution of benefits and risks) [25]. In addition, we evaluated the presence of ethical approval by an ethics committee to balance the benefits and risks to participants, future patients, or society. Additional publications found on the Influenzanet Consortium website [15] were gathered and screened for ELSIs as well. Furthermore, reference lists of included publications were searched for additional studies. Only publications mentioning at least one of these 6 ELSI components were included.

**Figure 1:**
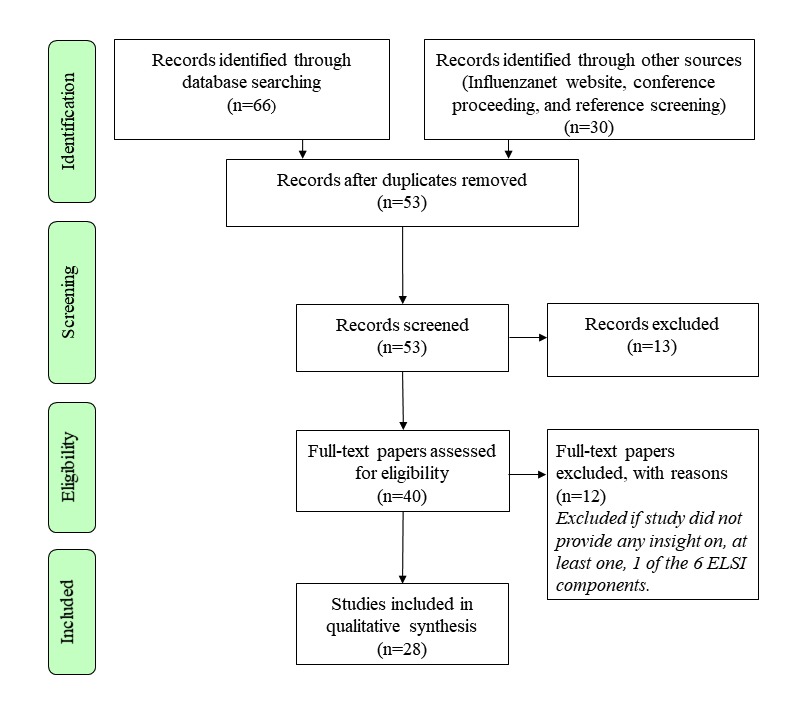
The PRISMA (Preferred Reporting Items for Systematic Reviews and Meta-Analyses) flowchart of study selection. ELSI: ethical, legal, and social issues.

The included full-text papers were screened and analyzed independently by 2 review authors (LDG and TW) to ensure that they met the inclusion criteria of having information on the desired ELSI components previously described. Discrepancies between the 2 review authors were solved through discussion. Figure 1 illustrates the methodological process behind the selection and inclusion of publications for this review based on the PRISMA framework for systematic reviews and meta-analyses [26]. When the gathered literature did not provide sufficient information on some national platforms, country members of the Influenzanet Consortium were contacted either through email or the coordinator of the Consortium to provide additional details and to assess the veracity of the information gathered on their respective platforms.

## Results

The original literature search, carried out on September 11 and repeated on October 10 2017 in the databases mentioned above, identified a total of 66 potentially relevant papers (Table 1) on the Influenzanet Consortium and its national platforms (eg, *De Grote Griepmeting* for the Netherlands and Belgium, *Flusurvey* for the United Kingdom and Ireland, *Gripenet* for Portugal and Spain, *Grippenet* for France and Switzerland, *Hälsorapport* for Sweden, *Influmeter* for Denmark, and *Influweb* for Italy). No papers on Influenzanet were found on the Global Digital Library on Ethics and BELIT.

After the removal of duplicates (n=29), 37 papers were considered for this study. Among these 37, 4 were datasets, 5 included supporting information to other papers in the list (eg, tables and figures), 2 papers were in other languages (French and Swedish), and 2 were meeting abstracts; these 13 papers were excluded. The remaining 24 full-text papers were then screened for 6 ELSI components (informed e-consent obtained from participants; ability of participants to opt out from the study at any time; Web-based information on national platform and influenza; personal data protection measures, for example, anonymization and abiding to the national regulations on privacy, data collection, and treatment; open and nondiscriminative participation; and ethical approval by an REC or other competent entity). Only 16 of the 24 papers addressed some ethical, legal, and social components (eg, ethical approval by RECs, informed consent, etc).

Our search of the Influenzanet Consortium website [15] resulted in an additional 28 publications. After the removal of duplicates (n=14), 14 publications were considered for a detailed review of their ELSI components. Notably, 10 of the 14 papers addressed some ethical, legal, and social components.

**Table 1:** Initial search results (date of search: September 11, 2017 and October 10, 2017).

Search terms used		Results found in PubMed (n=22), n	Results found in all Web of Science databases (n=44), n
European network: Influenzanet		10	16
**National platforms**			
	De Grote Griepmeting	0	0
	Flusurvey	3	7
	Gripenet	1	1
	Grippenet	6	12
	Hälsorapport	0	2
	Influmeter	1	1
	Influweb	1	5

Overall, 2 additional publications (retrieved from reference screening and a conference proceeding) were included, leading to a total of 28 papers included in our analysis, as seen in Figure 1. Table 2 reports on these 28 papers and summarizes the presence or absence of the ELSI components for each paper reviewed. However, Table 2 should be interpreted cautiously because the presence of some ELSI components does not automatically apply to all country platforms in noncountry-specific publications.

The Influenzanet country-specific information in Table 3 was collated from publications gathered in the literature search (Table 2) and additional information provided by country representatives. The information in Table 3 was subsequently double checked, updated, and corrected by country representatives (in many cases, authors of papers themselves) through the help of the supervisor of the Consortium who communicated our findings. This verification step was important to prevent inaccuracies resulting from the misinterpretation of the literature because the absence of an ELSI component in a publication does not automatically imply that it was not addressed by the platform(s) and temporal evolution of these platforms, where ELSI components might change over time.

Only 3 of 11 national platforms (Belgium-the Netherlands and Denmark) did not seek ethical approval by REC before the launch of their platforms. The Swiss national platform [45] has obtained ethical approval for the launch of its mobile app before the start of the flu season 2017/2018. Registration and participation to the national platforms were open and nondiscriminative to all residents of the respective countries [12,14], except for the Swedish platform, where participation is through invitations only [17,18]. The Web-based information on the study was provided to all Influenzanet participants and informed electronic consents were obtained from participants in 9 of 11 national Influenzanet platforms. The electronic consent or so-called “e-consent” commonly used in studies without face-to-face contact, but where communication is entirely taking place via Web-based technologies, is another exception to, or adaptation of, traditional informed consent. Influenzanet uses this type of e-consent, where participants through a few clicks on a screen agree to the terms, conditions, and privacy policies of the research project.

The Belgium-Dutch and Danish platforms were the only exceptions where an informed e-consent was not legally required for participation (Influmeter, email communication, August 10, 2017) [6,20,21]. All Influenzanet users were allowed to withdraw from the research at any time. Participant identifiers used in Influenzanet (eg, pseudonyms and email addresses) were stored separately from the questionnaire data and not used during the data processing and analysis phases. Personal data from participants were, thus, anonymized before processing or analysis, which were performed at the aggregate level in all national platforms [14].

**Table 2:** A list of included studies (n=28) with ethical, legal, and social issue components (with ethical approval of study). All platforms listed by each paper do not satisfy the ethical, legal, and social issue components equally.

Author (year)	Platform(s) concerned	Ethical, legal, and social issue components					
		Research ethics committee	Open and non-discriminative participation	Web-based information sheet	Informed e-consent	Opt out from study	Personal data protection measures
Adler et al (2014) [27]	UK^a^	✓	✓				✓
Bajardi et al (2014) [28]	BE^b^, FR^c^, IT^d^, NL^e^, PT^f^, SE^g^, UK	✓	✓	✓	✓		✓
Bajardi et al (2014) [29]	BE, FR, IT, NL, PT, SE, UK	✓	✓		✓		✓
Brooks-Pollock et al (2011) [19]	UK	✓	✓	✓	✓		✓
Cantarelli et al (2014) [14]	BE, FR, IT, NL, PT, SE, UK	✓	✓	✓	✓		✓
Debin et al (2013) [30]	FR	✓		✓			✓
Debin et al (2014) [10]	FR	✓			✓		
Eames et al (2012) [31]	UK		✓				✓
Eames et al (2012) [32]	UK	✓	✓			✓	✓
Friesma et al (2009) [5]	BE, NL		✓	✓			
Guerrisi et al (2016) [33]	BE, FR, IT, NL, PT, UK		✓	✓			✓
Kjelso et al (2016) [20]	DK^h^		✓			✓	
Koppeschaar et al (2017) [17]	BE, DK, FR, IE^i^, IT, NL, PT, SE, ES^j^, UK	✓	✓	✓	✓		✓
Land-Zandstra et al (2016) [34]	BE, NL		✓	✓			
Loubet et al (2016) [35]	FR	✓		✓	✓	✓	✓
Loubet et al (2016) [36]	FR	✓		✓	✓	✓	✓
Marquet et al (2006) [21]	NL		✓	✓			✓
Paolotti et al (2010) [37]	IT		✓	✓			
Peppa et al (2017) [38]	UK	✓	✓		✓		
Perrotta et al (2017) [39]	IT		✓				✓
Perrotta et al (2017) [40]	IT		✓				✓
Pini et al (2017) [18]	SE					✓	✓
Smolderen et al (2007) [41]	NL		✓				✓
Tilston et al. (2010) [42]	UK	✓	✓	✓			
van Noort et al (2007) [11]	BE, NL, PT		✓	✓			
van Noort and Stollenwerk (2008) [43]	BE, IT, NL, PT		✓			✓	
van Noort et al (2015) [9]	BE, IT, NL, PT	✓		✓			
Vandendijck et al (2013) [6]	BE		✓			✓	✓

^a^UK: United Kingdom.

^b^BE: Belgium.

^c^FR: France.

^d^IT: Italy.

^e^NL: the Netherlands.

^f^PT: Portugal.

^g^SE: Sweden.

^h^DK: Denmark.

^i^IE: Ireland.

^j^ES: Spain.

**Table 3:** The ethical, legal, and social dimensions of Influenzanet national platforms (with research ethics approval of study).

National platform	Date of creation	Research ethics approval of study	Open and non-discriminative participation	Web-based information sheet	Informed e-consent	Ability to opt out from the study	Personal data protection^a^
Belgium (Flanders)	2003	—^b^	✓	✓	—	✓	✓
Denmark	2013	—^c^	✓	✓	✓^c^	✓	✓
France	2012	✓	✓	✓	✓	✓	✓
Ireland	2013	✓	✓	✓	✓	✓	✓
Italy	2008	✓	✓	✓	✓	✓	✓
Portugal	2005	✓	✓	✓	✓	✓	✓
Spain	2012	✓^d^	✓	✓	✓^d^	✓	✓^e^
Sweden	2011	✓	—	✓	✓	✓	✓
Switzerland^f^	2016	✓	✓	✓	✓	✓	✓
The Netherlands	2003	—	✓	✓	—^g^	✓	✓
United Kingdom	2009	✓	✓	✓	✓	✓	✓

^a^Data anonymization before processing and analysis.

^b^Not applicable.

^c^Influmeter, email communication, August 10, 2017-September 07, 2017.

^d^GripeNet Spain, email communication, August 10, 2017.

^e^Information obtained from the Spanish national platform [44].

^f^Swiss Influenzanet platform [45]. Grippenet Switzerland, email communication, September 12, 2017.

^g^De Grote Griepmeting The Netherlands, email communication, January 10, 2018.

## Discussion

### Principal Findings

To the best of our knowledge, this is the first scoping review examining the similarities and differences in the implementation of research ethics for the country platforms of Influenzanet. Our comparative tables highlight the need for further clarification and harmonization of these ethical issues pertinent to citizens engaging in the digital disease surveillance across the Consortium. A number of ELSIs are similarly organized in the Consortium, for instance, participation is open and nondiscriminative to all residents of these countries (except for Sweden for representativeness and comparison purposes [17]), study information is provided to all participants, and they are free to opt out from the study. However, a number of ELSIs are also addressed differently; for instance, REC approvals and informed e-consent were sought for recruiting participants and collecting their data in 8 of 11 and 9 of 11 platforms, respectively. The following sections of the discussion will highlight the discrepancies seen in the implementation of research ethics for different country platforms.

Overall, 8 platforms of the Consortium obtained REC approval before the start of their studies. However, it is not known how national RECs judged and approved their respective studies; for instance, they could have considered the gathered personal health-related data from participants to be fully anonymized for which no informed consent is required or considered studies to be human subject research. In the latter case, RECs would need to evaluate if the balance between potential benefits and risks for study participants is favorable and ensure that participants received adequate information on these risks and benefits. Our comparative table shows that all country platforms where REC approvals had been sought obtained informed e-consents from participants; this seems to indicate that these national RECs consider this type of citizen participatory research as human subject research and that e-consent is considered a valid form of consent in this context. The regional REC in Geneva approved the implementation of the Swiss platform and the launch of its mobile app as a data collecting tool. Considered as human subject research in Switzerland, a reader friendly informed e-consent is requested from potential participants. Because we did not have access to additional REC evaluations, further studies are needed to determine how RECs from different countries debated the ethical issues. It is also well known that national RECs, as well as RECs within the same country, may assess and balance risk-benefit ratios differently. These divergences concerning the evaluation of similar Influenzanet projects in different countries could interfere with the harmonization of ethical approaches. Thus, we suggest more transparency in terms of ethical issues related to this type of technology-driven public health research. For instance, project leaders of national Influenzanet platforms could publish a summary of how RECs evaluated and debated the ethical issues of their respective studies (eg, if their studies fall under the category of human subject research and, thereby, need informed consent procedures, etc). Such transparency could help to harmonize the ethical approaches to be adopted by the country platforms even further.

REC approvals were not sought for the Belgian-Dutch (*De Grote Griepmeting*) and Danish (*Influmeter*) platforms (Influmeter, email communication, August 10, 2017) [6,20,21]. However, their studies abided by their national legislation on privacy and personal data protection (Influmeter, email communication, August 10, 2017) [6,20,21,46]; for instance, the *De Grote Griepmeting* privacy regulation was approved by the Dutch Data Protection Authority [21]. According to the Belgian and Dutch legislations, these are observational studies because no physical or psychological intervention is intended on participants [6,21,47]. Concerning *Influmeter*, the Danish platform is exempted from the REC approval for the following reasons: the Danish Data Protection Agency does not consider emails exchanged between study participants and *Influmeter* to be sensitive personal information; there is an automatic implicit consent from study participants because of its voluntary nature even if sensitive information is gathered (eg, health and coarse-grained geographical data); and the data manager of *Influmeter*, *Statens Serum Institute* that hosts a large proportion of Danish health data [48] received a broad permission from the Danish Data Protection Agency (record number: 2008-54-0474), which covers the surveillance of infectious diseases and identifiable sensitive information gathered by *Influmeter* (Influmeter, email communication, September 7, 2017) [20].

Another ethical issue arises in Influenzanet owing to the ability of participants to record personal data on other household members (eg, the elderly persons and children). Gathering data on underrepresented age groups is important, specifically, when they are the ones most vulnerable to influenza in terms of morbidity and mortality [5]. However, it is difficult to verify whether these family members, in particular, are legally competent and could provide consent themselves, having expressed their will for their personal information to be recorded by the participating family member. It would be interesting to evaluate through future research whether RECs have considered this issue or have simply considered the data collected from other family members to be anonymous and, thus, not identifiable.

Informed e-consent was gathered from study participants from all platforms with the only exceptions being the Belgian and Dutch platforms, which are mirror websites of each other (*De Grote Griepmeting*, email communication, January 10, 2018). It can be argued that there is an automatic implicit consent for Belgian and Dutch participants because registration to the study is voluntary. The Belgium-Dutch platforms [16] might consider providing an informed e-consent option to their participants in an attempt to harmonize consent practices across the Consortium if ever they resume their activities in the future. However, we noted that it is not clear whether informed consent was legally necessary in Belgium (“Law on experiments involving the human subject” of May 2004) and the Netherlands because of the observational natures of their studies (*De Grote Griepmeting*, the Netherlands, email communication, January 10, 2018) [6,21,47]. Indeed, the Belgian and Dutch legislations acknowledge the need for informed consent for interventional studies because of the potential physical or psychological harm to participants [6,21,47], but the legislations do not clearly define how broad the category of observational studies is; for instance, a detailed questionnaire revealing some very personal information can be seen as an intervention in the Netherlands [49]. Nonetheless, it is important to understand the shift from typical physical or psychological harm seen in medical research to informational harm in public health research involving Web-based communities of volunteer citizens or big data (eg, data discrimination) with the latter having potential repercussions on the physical and mental states of study participants (eg, stigmatization and discrimination for health insurance coverage) [50,51].

Critics might say that e-consent gathered from Influenzanet participants does not represent valid informed consent because researchers cannot control that participants read and understood the information. Although information on studies is available on their respective websites, participants usually have to look at different sections of the website to gather pertinent information on the study (eg, its goals, privacy policies, etc), which is tedious and unlikely to be read in detail. The Swiss legislation requires researchers to ensure that participants have understood all the information (provided in the written information form) through personal contact. According to the *Ethics Guidelines for Internet-Mediated Research* by the British Psychological Society, a valid consent can be assumed if there is an information sheet defining the study objectives and exact nature of questions before filling in the questionnaire, including a check box at its beginning and end where participants can tick in to give their explicit consent [52]. In addition, the Society recommends using a proper wording for “I agree” statements to encourage participants to read the information sheet, which should also include their rights to withdraw from the study at any time in a user friendly manner [52]. The Consortium could follow these guidelines to enhance its e-consent procedures. It is important to ensure that participants agreed (by clicking on an “I agree” checkbox) to the terms, conditions, and privacy policies listed on the informed consent sheet before being allowed to fill in the questionnaire. Such measures should be taken to ensure the validity of the informed consent from participants and for harmonization purposes, a standard information sheet could be used throughout the Consortium.

Personal data protection was ensured throughout the Consortium because participant data are pseudonymized, that is, participants’ personal identifiers are replaced with pseudonyms. Furthermore, any data that are shared with the public through the Web portal are fully anonymized and highly aggregated [14]. As to the anonymized data shared with members of the Consortium, it can be aggregated or not depending on whether the national partner who owns the data agrees to the request. It is also worth noting that sharing of Influenzanet data with internal and external researchers should not pose *a priori* any legal barriers because the General Data Protection Regulation of the European Parliament and the Council will not apply to data being rendered anonymous, that is, the information is not linked to an identified or identifiable natural person (Recital 26) and shared for research and statistical purposes [53]. From information gathered on some Influenzanet platforms (ie, France and Switzerland), it appears that linked anonymization [54] is involved in ensuring data protection, whereby participants’ email addresses and pseudonyms are stripped from the gathered health-related data before analysis and stored separately. It will be difficult to conclude how challenging or easy it could be to reidentify participants directly or indirectly from specific combination of variables (which are anonymous if considered in isolation but permit the identification if several of them are combined), in particular for vulnerable groups where privacy risks are higher [48] or groups with rare variable entries, for instance, large household compositions with >6 persons (that only account for 2% of households in the European Union [55]).

Our results show that there is a need to harmonize consent requirements and practice throughout the Consortium because differences in the abovementioned national consent requirements could be the source of obstacles for the next generation of data collection, which may include collection and sharing of more sensitive data on a larger scale. Thus, these differences could hinder future productive cross-national collaboration, which will be detrimental to research and quality disease surveillance. The consent requirements and practice could be harmonized through international or European Union regulations. However, this could take some time; for instance, General Data Protection Regulation aims to harmonize data protection laws within the 28 European Union Member States to ensure an equivalent level of protection and freedoms of individuals within the European Union. At the same time, it will protect cross-border flows of personal data on European Union citizens to international organizations or third countries [56,57]. A quicker harmonization alternative would be for all national platforms of Influenzanet to use the strictest consent requirements and practice currently used by one of their platforms to find common ground.This particular platform would then be used as a benchmark for other country platforms to harmonize their practices.

### Limitations

Only English-language papers were screened for this study for practical reasons. It is, thus, possible that pertinent papers in other languages were omitted but they could have provided better insight into how issues related to research ethics are being handled by the Influenzanet Consortium. In addition, most of our conclusions are based on the gathered literature, and our interpretations might be biased by the incomplete reporting of all ELSI components in some publications as a matter of space or pertinance to their study objectives. Consequently, despite our collaboration with the Consortium to verify our claims, we cannot guarantee that our interpretations are error free. Another limitation is that we do not have access to projects’ evaluation reports from RECs (except for Switzerland) to understand how they judge and approve such research projects. This would have been beneficial to the understanding of national differences in project evaluation by RECs, which could then serve as the basis for further harmonization of these ethical approaches.

### Conclusions

Epidemics forecasting activities such as Influenzanet are beneficial. Harmonized criteria for dealing with ethical issues are urgently needed internationally to ensure comparability of data and maximize participants’ trust. Approaches used in handling issues related to research ethics by different Influenzanet platforms seem to be similar in many, although not all, ELSIs at present. Thus, harmonizing ethical requirements across the Consortium is feasible and could be achieved through the adoption of the strictest ethical requirements and practice currently used by one platform across the Consortium. Nonetheless, despite being similar, it does not automatically mean that these ethical approaches are adequately regulated. We recommend more transparency in terms of ethical issues related to this type of technology-driven public health research. These transparency modifications related to the current ELSIs of Influenzanet will help to build trust among the members of the general public, in particular, if they are properly informed about the expected benefits and potential risks their participation entails; this will prevent any decline in participation, which might be triggered by mediatization, including exaggeration, of the risks of public health surveillance using Web-based communities of volunteer citizens.

Moreover, this type of research has the potential to save many lives in the future because it has proven through its flexibility, easy implementation, and adaptability to different countries’ requirements for data collection to serve as a potentially effective and relatively low-cost surveillance tool for other diseases of public health importance (eg, Middle East respiratory syndrome or Ebola) [12,17]. These characteristics could allow Influenzanet to be deployed in low- and middle-income countries to monitor emerging and reemerging infectious diseases [17].

Our suggested harmonization measures and approach for data gathering and ethical requirements for Influenzanet apply to other Influenzanet-like systems. Moreover, we also suggest that such systems increase the validity of their informed e-consent procedures by following the excellent ethical guidelines provided by the British Psychological Society. Such measures will further increase the benefits Influenzanet and Influenzanet-like systems could bring to society by promoting the comparability of data and safeguarding participants’ trust on which they rely almost completely for data collection. These will ensure that Influenzanet-like systems are making even greater substantial contributions to global public health and reduce the health inequalities in societies worldwide through better targeting of public health interventions in line with the concept of *precision global health* in the digital age [58].

## Abbreviations

BELIT: Bioethics Literature Database
EISN: European Influenza Surveillance Network
ELSI: ethical, legal, and social issues
ILI: influenza-like illness
REC: Research Ethics Committee
